# Hydrophobic Modification of Pectin Aerogels via Chemical Vapor Deposition

**DOI:** 10.3390/polym16121628

**Published:** 2024-06-08

**Authors:** Eleni Effraimopoulou, Julien Jaxel, Tatiana Budtova, Arnaud Rigacci

**Affiliations:** 1 Mines Paris, PSL University, Centre for Processes, Renewable Energy and Energy Systems (PERSEE), 06904 Sophia Antipolis, France; eleni.effraimopoulou@minesparis.psl.eu (E.E.); julien.jaxel@minesparis.psl.eu (J.J.); 2 Mines Paris, PSL University, Centre for Materials Forming (CEMEF), UMR CNRS 7635, 06904 Sophia Antipolis, France

**Keywords:** porous materials, aerogels, pectin, chemical vapor deposition, methyltrimethoxysilane, composite, hydrophobicity, thermo-hydric aging, thermal conductivity

## Abstract

Pectin aerogels, with very low density (around 0.1 g cm^−3^) and high specific surface area (up to 600 m^2^ g^−1^), are excellent thermal insulation materials since their thermal conductivity is below that of air at ambient conditions (0.025 W m^−1^ K^−1^). However, due to their intrinsic hydrophilicity, pectin aerogels collapse when in contact with water vapor, losing superinsulating properties. In this work, first, pectin aerogels were made, and the influence of the different process parameters on the materials’ structure and properties were studied. All neat pectin aerogels had a low density (0.04–0.11 g cm^−1^), high specific surface area (308–567 m^2^ g^−1^), and very low thermal conductivity (0.015–0.023 W m^−1^ K^−1^). Then, pectin aerogels were hydrophobized via the chemical vapor deposition of methyltrimethoxysilane using different reaction durations (2 to 24 h). The influence of hydrophobization on material properties, especially on thermal conductivity, was recorded by conditioning in a climate chamber (25 °C, 80% relative humidity). Hydrophobization resulted in the increase in thermal conductivity compared to that of neat pectin aerogels. MTMS deposition for 16 h was efficient for hydrophobizing pectin aerogels in moist environment (contact angle 115°) and stabilizing material properties with no fluctuation in thermal conductivity (0.030 W m^−1^ K^−1^) and density for the testing period of 8 months.

## 1. Introduction

Aerogels are solid open-pore nanostructured materials that possess low bulk density, very high specific surface area and a lot of fascinating physical properties. Classical aerogels are made from a gel in which the liquid phase in the pores is replaced by air, using processes that minimize the capillary forces during liquid evacuation. As a result, the network morphology is preserved. Drying with supercritical carbon dioxide (scCO_2_) is the typical technique used for this purpose [1]. The first family of aerogels broadly studied were inorganic aerogels and, more specifically, silica aerogels. They hold typical densities in range of 0.05–0.30 g cm^−3^ and specific surface areas higher than 800 m^2^ g^−1^ [2]. It was then realized that these unique properties, along with the mesoporous structure of silica aerogels, make them exceptional thermal insulators with thermal conductivity far below that of air (0.025 W m^−1^ K^−1^) and as low as 0.013 W m^−1^ K^−1^ in ambient conditions [3,4]. This property range is named thermal superinsulation. Silica composite or hybrid aerogels, and also some synthetic polymer-based aerogels, were later studied with regard to their thermal insulating properties, the latter possessing thermal conductivity values equal or higher than the ones of neat silica [5,6,7,8,9,10].

The need of a greener approach for the design of advanced materials made essential the development of bio-based aerogels as more sustainable alternatives to conventional, non-renewable counterparts. Bio-based aerogels were developed at the lab scale in the past two decades. It was shown that in some cases, they can exhibit the characteristics of conventional aerogels such as low density and high specific surface area, as well as mesoporosity, and also possess the characteristics specific to bio-based polymers such as biodegradability and often biocompatibility. A wide range of bio-aerogels were obtained based on polysaccharides such as cellulose [11,12,13], chitosan [14], starch [15,16], alginate [17,18], pectin [19,20,21], proteins [22,23], or other polymers extracted from biomass [24]. Bio-aerogels are very attractive materials for a very wide variety of applications. These include thermal insulators [25], acoustic insulators [26], batteries’ electrodes [27], supercapacitors [28], piezoelectric transducers [29], CO_2_ captures [30], absorbents [31], drug carriers [32], catalyst supports [33], and food packaging [22]. Several bio-aerogels were studied for their thermal insulation properties, and materials based on nanocellulose, starch, and pectin were shown to fall in the superinsulating area [16,21,34,35]. Among them, pectin aerogels possess density around 0.1 g cm^−3^, specific surface area up to 600 m^2^ g^−1^ and thermal conductivity in ambient conditions as low as 0.015 W m^−1^ K^−1^, which is very close to the thermal conductivity of the best silica aerogels [21].

Pectin is a polyelectrolyte with a complex structure mainly consisting of D-galacturonic acid (GalA) units that are connected by a-(1–4) glycosidic linkages. These acids have carboxyl groups, some of which are methyl esterified. Commercially available pectin is derived from citrus peel or apple pomace from the waste of the juice manufacturing industry [36,37]. Pectin is used predominantly for food applications due to its gelation and thickening properties. The gelation of pectin depends on various parameters such as the degree of esterification (DE), the temperature, polymer concentration and pH of the solution, the presence of salts and/or sugars, molecular weight, etc. The processing parameters define the gelation kinetics and the gel morphology. Two common ways to achieve pectin gelation are known: acidic gelation or crosslinking with polyvalent metal ions [38,39]. Another method to obtain pectin networks is by non-solvent-induced phase separation [20,21]. The formation of the pectin network plays a crucial role in the final morphology of the dry material and hence the material properties [20,21].

If willing to use bio-aerogels for the engineering (thermal or acoustic insulation) applications, their aging properties in different environments should be investigated. Here, the disadvantage of bio-aerogels (including pectin aerogels) is the high intrinsic hydrophilicity of the polymer and, thus, of the final porous material. The researchers working with bio-aerogels know well that water vapors from the atmosphere trigger the collapse of the fine porous network of a bio-aerogel and irreversibly alter their properties. However, there have been very few reports on the aging of bio-aerogels, and, in particular, on the evolution of their thermal conductivity in humid conditions. For example, an almost two-fold increase in the thermal conductivity (from around 0.04 to around 0.08 W m^−1^ K^−1^) was recorded for nanocellulose foams when the relative humidity (RH) increased from 20 to 80 RH% at room temperature [40]. An even higher conductivity increase (from around 0.027 to 0.120–170 W m^−1^ K^−1^ at 0 to 60 RH%, respectively) was reported for porous cellulose made by dissolution in aqueous NaOH solvent, coagulation and freeze drying [41]. In the latter case, plasma treatment of the porous dry material allowed cellulose hydrophobization and stabilization of thermal conductivity around 0.030–0.033 W m^−1^ K^−1^ for 24 h at 60 RH%.

In general, several physical or chemical modifications are proposed in the literature for the hydrophobization of polysaccharides or polysaccharide-based materials [42,43,44,45,46]. Physical modifications include plasma technologies [47,48] or the adsorption of hydrophobic molecules/macromolecules [49]. Chemical modifications are silylation [50,51,52], esterification [53,54], etherification [55], etc. Modifications can be applied to the starting polymer as a homogeneous approach or to the material surface (or pores’ surface) as a heterogeneous approach. For example, various modifications have been applied to make freeze-dried cellulose for absorption/adsorption and separation applications (see, for example [48,56]). Silylation was applied to nanocellulose aerogels to make the material hydrophobic [57]; however, no evolution of thermal conductivity in time was reported. No hydrophobic pectin aerogels have been reported, and nothing is known about the evolution of pectin aerogel thermal conductivity in time in different RH conditions.

The main objective of this work was to study the evolution of pectin aerogel properties as a function of time in high relative humidity conditions and suggest a chemical modification which should keep thermal conductivity stable in time and below 0.030 W m^−1^ K^−1^ in room conditions. The novelty of this work consists of (i) using the chemical vapor deposition (CVD) of methyltrimethoxysilane (MTMS) for pectin aerogel modification and (ii) in finding conditions for the efficient stabilization of thermal insulation properties. This type of modification was chosen as a more environmentally friendly procedure since it does not use any organic solvents. First, reference neat pectin aerogels were synthesized, and the influence of the main processing parameters was correlated with the material properties. Then, CVD was applied to selected pectin aerogels for 24 h, and the evolution of thermal conductivity of neat and silylated pectin aerogels as a function of time at high RH (80%) was monitored. Finally, material optimization was performed by adjusting CVD duration. It was demonstrated that the introduction of methyl silyl groups is very efficient for pectin aerogels’ hydrophobization. ensuring a long-term stability of the thermal insulation performance.

## 2. Materials and Methods

### 2.1. Materials

Apple pectin with degree of esterification (DE) 50–75% (as defined by the provider) was purchased from Sigma Aldrich (Darmstadt, Germany). The actual DE (%) and molecular weight were determined using titration and viscometry measurements, respectively (see Section 2.2.1). Hydrochloric acid (HCl) (37%, laboratory reagent grade), potassium hydroxide pellets (KOH) (laboratory reagent grade), absolute ethanol (purity > 99%), acetone (purity > 99%, laboratory reagent grade) and ammonium hydroxide (28–30% solution in water, ACS reagent) were obtained from Fisher Scientific (Illkirch-Graffenstaden, France) and sodium hydroxide flakes (NaOH) (98% purity) were purchased from Alfa Aesar (Illkirch-Graffenstaden, Germany). Methyltrimethoxysilane (MTMS) (purity > 99%, reagent grade) was from Sigma-Aldrich (Darmstadt, Germany). 

Solvents and reagents were used without further purification. Water was deionized (DI).

### 2.2. Methods

#### 2.2.1. Characterization of Pectin

The degree of esterification (DE) and pKa of the pectin were determined by potentiometric titration [58]. The DE corresponds to the molar proportion (%) of methoxy groups in the pectin and is given by Equation (1):

(1) DE=KeKt×100=(Kt−Kf)Kt×100=1−KfKt×100

where *K_e_* is the number of esterified carboxyl groups, *K_f_* is the number of free carboxyl groups, and *K_t_* is the total number of carboxyl groups, i.e., *K_t_* = *K_f_* + *K_e_*.

To determine the amount of free carboxyl groups (*K_f_*), pectin (0.2 g) was dissolved in DI water (20 mL) and was titrated with a 0.1 M KOH solution. The titration was repeated for three independent samples, and the mean value was considered. 

The total amount of carboxyl groups, *K_t_*, was determined as follows. A 0.1 M KOH solution was used to neutralize the pectin sample after determination of the free carboxyl groups. Ten milliliters of 0.1 M KOH were further added, and the solution was stirred at 40 °C for 4 h to saponify the esterified carboxy groups of the polymer. Afterwards, 10 mL of 0.1 M HCl was added to protonate the carboxylate moieties into carboxyl acid moieties. The excess of HCl was then titrated with 0.1 M KOH.

The obtained *K_f_* and *K_t_* values allow the calculation of the DE of pectin that was 58 ± 2%. The pK_a_ of the pectin was 3.0.

Viscometry and the Huggins approach were used to determine pectin intrinsic viscosity [η] using iVisc from LAUDA (Lauda-Königshofen, Germany) and a capillary Ubbelhode Dilution Viscometer Type I with a capillary diameter 0.63 mm. The solvent was 0.01 mol L^−1^ NaCl, and the measurements were performed at 26.6 °C [59]. The molecular weight M_η_ was calculated using the Mark–Houwink equation:

(2) $$[\eta ]\;=K\;M_{\eta }^{\alpha }$$

where *K* = 0.0234 and *α* = 0.8221 [59]. The intrinsic viscosity of pectin in these conditions was 342 mL g^−1^, and the molecular weight was found to be equal to 1.16 × 10^5^ g mol^−1^.

#### 2.2.2. Preparation of Pectin Aerogels

**Figure 1:**
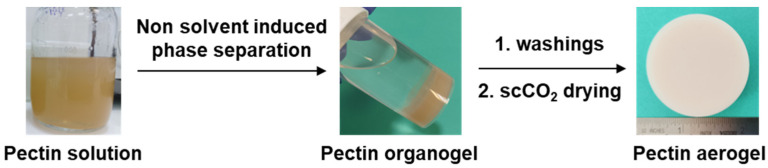
Preparation process of pectin aerogels.

Pectin aerogels were prepared via the dissolution of pectin in water followed by the non-solvent-induced phase separation approach [20,21]. Pectin concentrations in the starting solution were 2 and 3 wt%, and pH values were adjusted to 2 and 3 by the addition of a small quantity of HCl or KOH, respectively. The following formulations 2 wt%/pH 2; 2 wt%/pH 3; 3 wt%/pH 2 and 3 wt%/pH 3 were prepared and will be noted (X, Y) with X corresponding to pectin concentration and Y corresponding to solution pH. The pectin solution was then poured into molds of 27.5 mm diameter, and the solvent-to-non-solvent exchange was performed. Two pectin non-solvents were used: ethanol (E) and acetone (A) [20]. Solvent/non-solvent exchange was performed progressively with the water/non-solvent ratio (*v*/*v*) decreasing to 50/50, 25/75, and 0/100, which was followed by a final extensive washing with pure non-solvent. This procedure led to a fully coagulated pectin network forming a monolithic and self-standing organogel (Figure 1).

The obtained eight formulations of organogels were dried with supercritical CO_2_ drying to result in pectin aerogels named (X, Y, E) or (X, Y, A). Supercritical CO_2_ (scCO_2_) drying was performed by placing pectin organogels into a 1 L autoclave. The system was closed and pressurized at approximately 40 bar and 37 °C with gaseous CO_2_. Then, the pressure was increased to 80 bar. A dynamic washing step (at 50 g min^−1^) at 80 bars and 37 °C was carried out for 4 h. Afterwards, the system was slowly depressurized overnight at 4 bar/h and 37 °C and cooled down to room temperature by inertia. The autoclave was then opened, and the samples were collected.

Due to the intrinsic hygroscopicity of pectin, all pectin aerogels were immediately placed into desiccators to protect them against moisture adsorption. 

The aging of pectin aerogels was performed in a Binder KBF 115 climatic chamber conditioned at 25 °C and RH of 80%.

#### 2.2.3. MTMS Chemical Vapor Deposition on Pectin Aerogels

**Figure 2:**
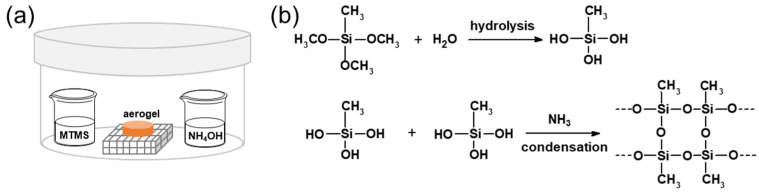
Schematic representation of the set-up for MTMS vapor deposition on pectin aerogels (**a**) and reaction of MTMS polymerization inside the pores of pectin aerogels (**b**).

Pectin aerogels were placed in a 5 L chamber (Figure 2). Five grams of MTMS and five grams of NH_4_OH were placed in the chamber in separate containers, avoiding physical contact with the aerogels (Figure 2). The chamber was tightly closed and left at room temperature for 2, 5, 16, or 24 h [60]. Finally, the chamber was opened, and the samples placed into desiccators.

#### 2.2.4. Characterization of Pectin Aerogels

Volumetric shrinkage of samples at each processing step was determined by measurement of the materials’ dimensions with a digital caliper (±0.01 mm) before and after the step (Equation (3)).

(3) Volumetric shrinkage, % =Vi- VfVi× 100

where *V_i_* is the volume of the material before the specific step and *V_f_* is the volume after. The maximal error was 8%.

The bulk density, *ρ_b_*, was determined as the ratio of sample mass to volume, the former measured with a digital analytical balance with a precision of 0.01 mg and the latter using a high-precision caliper. The maximal error in density was 0.01 g cm^−3^.

The porosity of the neat pectin aerogels was estimated from bulk and skeletal densities as follows (Equation (4)).

(4) $$\Pi \;\%=(\rho_{s}\;–\;\rho_{b})/\rho_{s}\times 100$$

with *ρ_s_* being the skeletal density of 1.57 g cm^−3^ [19].

ATR-FTIR spectra were obtained with a Bruker Tensor 27 equipped with a PIKE MIRacle™ singe reflection accessory, using OPUS 7.8 software. The measurements were performed in the range of 4000–600 cm^−1^ with a resolution of 4 cm^−1^ and 16 scans per analysis.

The specific surface area was determined by nitrogen adsorption either using ASAP 2020 (Micromeritics, Mérignac, France) or Belsorp mini X (Microtrac, Toulouse, France) and a Brunauer–Emmett–Teller (BET) model. Prior to analysis, samples were degassed in a high vacuum at 70 °C for 10 h. The average error was around 15 m^2^/g and the maximal one was 35 m^2^/g.

Scanning electron microscopy (SEM) observations and energy-dispersive X-ray (EDX) analysis of aerogel inner morphology and composition, respectively, were performed using Tescan (Fuveau, France) MAIA3 XMU. Prior to observations, a fine layer of 7 nm of platinum was sputtered onto the sample surface with Q150T Quarum (Lewes, UK) rotating metallizer.

Water contact angle measurements were performed with a Krüss (Hamburg, Germany) DropAnalyzer DSA 100 goniometer and calculated using Drop Shape Analysis v1.9-02 software. The maximal error was 1°.

The thermal conductivity (*λ*) of pectin aerogels was measured at ambient pressure using a heat flow meter LaserComp Fox 150 equipped with a custom “micro-flow meter cell” developed for small samples [19,21,61], typically cylinders of 20 mm in diameter, at 20 °C. Spaceloft^®^ aerogel from Aspen (thickness of 3.70 mm) with thermal conductivity of 0.0133 W m^−1^ K^−1^ at 20 °C according to the European Norm EN 12667 [62] was used as a standard for calibration. The maximal error was 0.0005 W m^−1^ K^−1^.

Moisture uptake by pectin aerogels was calculated by measuring the samples’ mass at the specific time interval with a digital analytical balance and a sample volume with a digital caliper. The maximal error was 2%.

## 3. Results

First, an overview of neat (non-hydrophobized) pectin aerogels is presented, as they will be used as reference materials. For more details on the influence of processing conditions on the pectin aerogels’ structure, properties and thermal conductivity, the reader is advised to look in refs [20,21]. Next, the properties of composite aerogels obtained after 24 h deposition of MTMS are reported. Finally, the influence of the duration of MTMS deposition on the composite aerogel properties, focusing on aging in the climatic chamber, is presented and discussed.

### 3.1. Properties of Neat Pectin Aerogels

As pectin is a polyelectrolyte, solution pH is very important to understand polymer interactions. At very low pH values, pectin chains are stabilized by both intra- and interchains’ hydrogen bonding between the protonated carboxylic acid groups and the alcoholic groups of the polysaccharide chains and by hydrophobic interactions of the methyl esterified groups. The concentration of the polymer in the solution affects the ability to form sufficient interactions that will lead to gelation [38,39]. For the studied pectin concentrations (either 2 wt% or 3 wt%) and solution pH (either 2 or 3), pectin was fully dissolved in water, and acidic gelation did not occur. This observation is in accordance with the literature [20]. When a pectin non-solvent was added in the system, pectin coagulated, forming a 3D network. The non-solvent nature along with the polymer concentration and pH of the starting pectin solution had an impact on the network structure and resulted in materials with different morphologies and properties. They are summarized in Table 1 and Table S1 and presented graphically in Figure S1.

During solvent exchange and drying, all neat pectin aerogels exhibited a high total volumetric shrinkage (64–82%, Table S1). During the solvent exchange step, the volumetric shrinkage was higher for the samples prepared with acetone as a non-solvent. For all the samples, the highest volumetric shrinkage occurred during the drying procedure (57–82%). Here, organogels prepared from solutions at pH 3 and with ethanol as non-solvent exhibited the greatest shrinkage values. Overall, the total shrinkage was slightly lower for solutions made at pH 2. This behavior was expected, as a larger number of hydrogen bonds were formed at lower solution pH, helping pectin to “resist” from strong collapse. In all cases, the final materials had low bulk densities (0.04–0.11 g mL^−1^), which is slightly higher for the samples prepared at pH 3 (Table 1), as a result of higher shrinkage during the fabrication process. The BET surface areas were in the range of 308–556 m^2^ g^−1^ (Table 1). The highest value, 556 m^2^ g^−1^, was obtained for the pectin aerogels prepared from the formulation (3, 2, A).

All neat pectin aerogels were thermal superinsulating materials with thermal conductivity in the range of 0.0148–0.0234 W m^−1^ K^−1^, i.e., below that of air in ambient conditions (0.025 W m^−1^ K^−1^) (Table 1). The lowest conductivity was recorded for aerogels with density values around 0.08 g cm^−3^ corresponding to formulations (2, 3, A) and (3, 3, A) (Table 1). Similar low values of thermal conductivity at similar density were reported for pectin of DE 35% [21].

All neat pectin aerogels exhibited excellent thermal conductivity values that make them suitable for thermal insulation materials. However, pectin is a water-soluble polymer and is aging with time due to the adsorption of water vapors, as will be shown below. Hydrophobization by CVD of MTMS was thus performed to try to prevent material aging.

### 3.2. Properties of Composite Pectin Aerogels after 24 h of MTMS Vapor Deposition

The modification of pectin aerogels was performed using MTMS as a hydrophobic agent and NH_4_OH as the reaction catalyst [60]. MTMS and ammonia were placed in a closed container together with the pectin aerogels, as shown in Figure 2. All neat pectin aerogels (i.e., made from solutions of concentration 2 wt% or 3 wt%, at pH 2 or 3, and non-solvent ethanol or acetone) were kept in MTMS vapor phase for 24 h. As it will be shown in the following, vapors of MTMS diffused into aerogel pores and, in the presence of water and NH_3_, MTMS polymerization occurred (Figure 2b), resulting in a silica-based phase that was deposited around the pectin fibrils of the bio-aerogel network. The chemical composition and material properties of the final composite aerogels are presented below.

**Figure 3:**
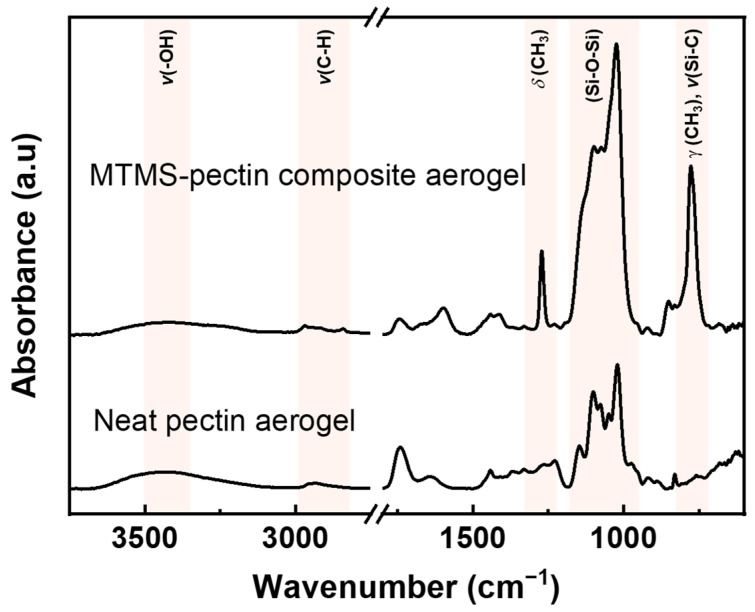
ATR-FTIR spectra of neat pectin aerogel and MTMS–pectin composite aerogel obtained after 24 h reaction. The characteristic bands of the deposited silica-based polymer are marked on the spectra.

The chemical modification of pectin aerogels was confirmed using ATR-FTIR spectroscopy performed on the interior of the specimens once cut (compare spectra for neat pectin and for composite aerogels after 24 h reaction in Figure 3). The spectrum of neat pectin aerogels showed all characteristic peaks of pectin [63]. The broad band at 3400 cm^−1^ was assigned to the stretching vibrations of the –OH groups of the pectin chain and water molecules that were inside the material’s network. The bands that appear around 2930 cm^−1^ were assigned to the C-H stretching of the –CH groups of the ring. Two bands were observed corresponding to C=O stretching vibrations at 1739 cm^−1^ and 1641 cm^−1^. These bands were assigned to the methyl esterified groups and the carboxylate groups, respectively. A weaker band corresponding to the C=O stretching of the ionic carboxyl groups appeared at 1440 cm^−1^. The multiple bands in the area of 930–1200 cm^−1^ were attributed to the glycosidic bonds, and the sharp band at 1020 cm^−1^ was assigned to the C-O stretching on the saccharide ring. After modification, most of the pectin’s characteristic bands were not visible, as they were overlapping with the strong bands coming from the silica-based polymer. The intensity of the band around 3400 cm^−1^ attributed to the –OH groups of pectin or water molecules decreased. This was a result of the interactions between the pectin –OH groups and the silica-based polymer and the decrease in the amount of water molecules inside the material matrix due to the hydrophobic nature of the modification. Additional bands around 2930 cm^−1^ were observed for the MTMS-modified pectin, corresponding to the C-H stretching of the Si-CH_3_ groups. Around 1047–1137 cm^−1^ strong bands appeared corresponding to the stretching and bending vibrations of the Si-O-Si group. The bands at 777 cm^−1^ and 1273 cm^−1^ were assigned to the –Si-CH_3_ groups and were correlated with the CH_3_ symmetric deformation and Si-C stretching, respectively. All characteristic bands of the deposited silica polymer were observed as reported in the literature [64,65].

**Figure 4:**
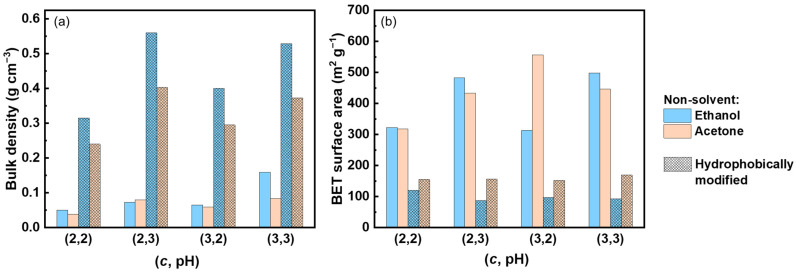
Comparison of bulk density (**a**) and BET surface area (**b**) of neat pectin aerogels and with composite aerogels obtained for reaction time of 24 h.

The properties of composite aerogels obtained after 24 h reaction are summarized in Figure 4, and the values are provided in Table S2. After 24 h reaction, a significant amount of mass increase (around 150–200 wt%) occurred for all materials, and a volumetric shrinkage was observed (Table S1). The volumetric shrinkage during the reaction was higher for the aerogels prepared with ethanol as non-solvent (35–46%) compared to that in acetone (18–28%). All composite aerogels showed higher density values in comparison with the neat pectin aerogels (Figure 4a), which is in accordance with the mass increase and volumetric shrinkage. The lowest density was obtained for the composite aerogels prepared from solutions of pectin concentration 2 wt% and pH 2 and non-solvent acetone, i.e., (2, 2, A) formulation. It is noted that this formulation possessed the lowest density for the neat pectin aerogels as well. Densification of the network during modification resulted in lower values of BET surface area for all modified aerogels (Figure 4b) with the lowest values for composite aerogels prepared with ethanol as non-solvent (87–129 m^2^ g^−1^) and the highest values, very close to each other, for the composite aerogels prepared with acetone as non-solvent (152–169 m^2^ g^−1^).

**Figure 5:**
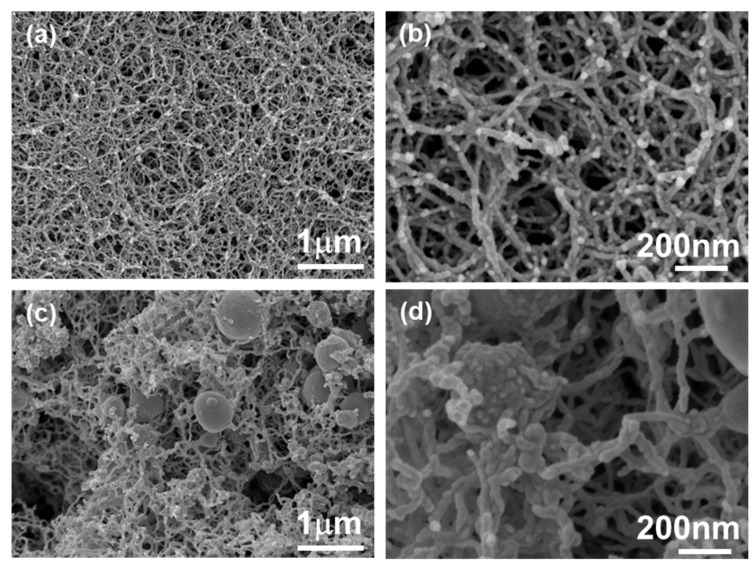
SEM images of (**a**,**b**) neat pectin aerogels and (**c**,**d**) corresponding composite aerogels after the reaction during 24 h. Pectin aerogels were from the formulation (2, 2, A).

The internal morphology of the composite aerogels obtained after 24 h reaction was analyzed with SEM (Figure 5). All neat pectin aerogels were materials with a fine nanostructured network of fibrils, as has been observed for pectin aerogels before [19,20,21]. The composite aerogel network maintained the fibrous nanostructure of their parent counterpart. Then, 24 after the reaction, the fibrils became thicker, which was in accordance with the deposition of silica polymer on the already formed and dried pectin fibrils. As revealed from the SEM images, the silica-based polymer not only formed a “coating” around pectin fibrils but also particles that were dispersed inside the fibrous network. These particles appeared to be non-porous, as observed by SEM, which is in the agreement with the decrease in surface area values of composite aerogels compared to that of neat pectin counterparts (Figure 4b). Consequently, the network was denser for the composite aerogels, which was in accordance with the bulk density values (Figure 4a).

**Figure 6:**
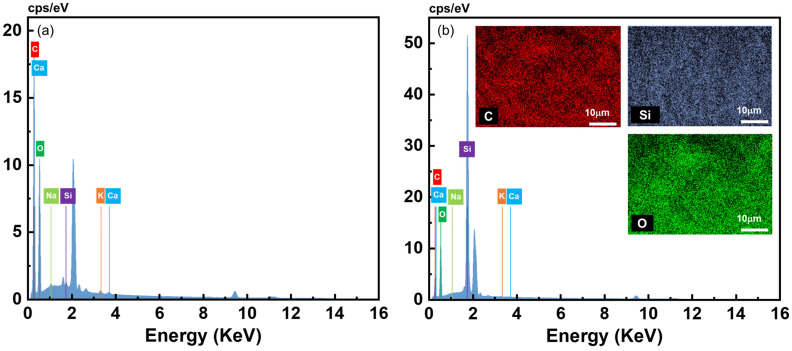
EDX mapping of (**a**) neat pectin aerogel, formulation (2, 2, A) and (**b**) of internal area of the corresponding composite aerogel (reaction duration 24 h). The peak at 2.05 corresponds to Pt that is used for sputtering.

The composition of the neat and composite pectin aerogels was analyzed using EDX spectroscopy (Figure 6 and Table 2). This method provides elements’ mapping and concentration in a selected area. EDX analysis of neat pectin aerogels (Figure 6a, Table 2) revealed, as expected for a carbohydrate, a high content of carbon and oxygen atoms, with their proportion in agreement with data from the literature [66], and with a negligible concentration of silicon. After the modification with MTMS for 24 h, the mass percentage of silicon increased to 24% in the mapped area (Figure 6b, Table 2). For this formulation, (2, 2, A), the mass increase during the reaction was 154%, as shown in Table S2. The distribution of carbon, oxygen and silicon on the selected inner surface of the composite aerogel shows a homogeneous distribution of all elements and, in particular, of the silica-based polymer (Figure 5). EDX results confirmed that MTMS diffused inside the pectin network and was deposited on the aerogel pore walls.

**Figure 7:**
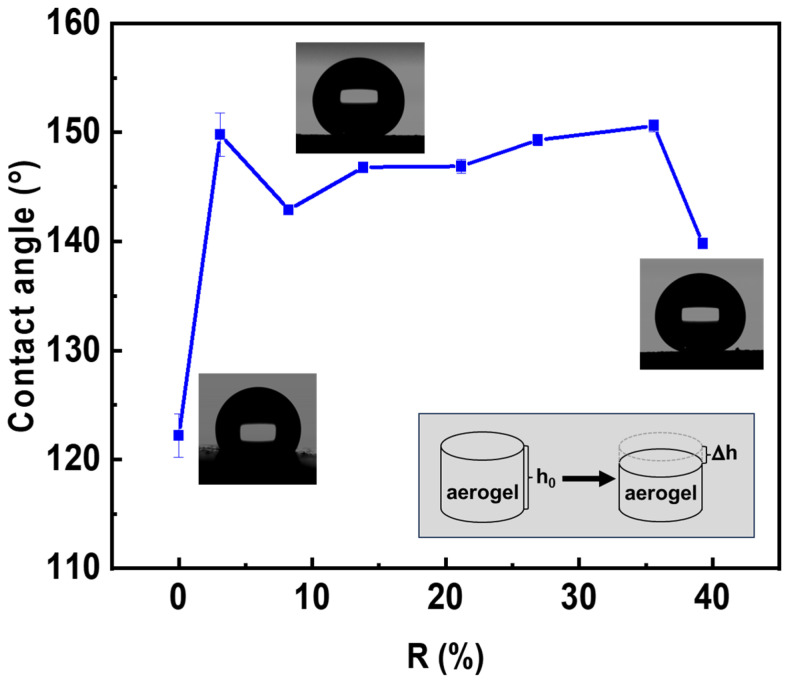
Water contact angle of composite aerogel at different depths inside the sample. Pectin aerogel was made from (2, 2, A) formulation, and the duration of MTMS vapor deposition was 24 h.

All composite pectin aerogels modified during 24 h by MTMS vapors were evaluated toward material hydrophobicity by measuring the contact angles with water. In accordance with the hydrophobic nature of the silica-based polymer that covered the hydrophilic pectin fibrils, all composite aerogels were hydrophobic with water contact angles in the range of 116–127° (Table S2). Water contact angles for neat pectin aerogels were not recorded, as droplets were absorbed immediately (less than one second), making the analysis impossible. To check if hydrophobization occurred within the whole body of composite aerogels, parts of the sample were gradually removed, and the contact angle with water was measured. Figure 7 shows the contact angles at various “depths” R in the sample:

$$R,\;\%=\frac{∆h}{h_{0}}\times 100\%$$

where Δ*h* is the thickness of the removed layer and *h*_0_ is the initial thickness of the sample. Contact angles of 140–151° were obtained for the interior of the sample, which was down almost to half of the initial thickness of the composite aerogel (*R* = 40%). Thus, the hydrophobic modification was equally successful inside the aerogel and on its surface. The increase in the water contact angle in the interior of the composite aerogels compared to its surface could be attributed to the increase in roughness due to mechanical material removal.

### 3.3. Influence of Reaction Time of MTMS Chemical Vapor Deposition on Composite Aerogel Properties

In the previous section, the properties of hydrophobically modified pectin aerogels via the vapor deposition of MTMS over 24 h were presented. To achieve materials with very low thermal conductivity in ambient conditions and, especially, superinsulating materials, the first basic requirement is that the material density should be around 0.1 g cm^−3^, as demonstrated by numerous studies [25]. Modification with MTMS for 24 h resulted in materials with bulk densities in the range of 0.24–0.53 g cm^−3^ (Figure 4); in addition, non-porous silica-based micron-size particles inside the pectin network were formed, deteriorating the composite aerogel properties in terms of the morphology and specific surface area. These features made the composite aerogels obtained via MTMS vapor deposition for 24 h not suitable for thermal insulation applications. Thus, the hydrophobization reaction time was varied to achieve hydrophobic aerogels with significantly lower bulk density values and without non-porous particles inside. Three different vapor deposition times were tested for this purpose, 2, 5, and 16 h, keeping all the other reaction parameters (temperature, pressure, etc.) constant. Since the goal was to reach the lowest possible density values (while maintaining the hydrophobic property), the formulations that were selected were the ones that resulted in the lowest bulk density for both neat and chemically modified pectin aerogels: these are pectin solutions that were coagulated in acetone, and, in particular, (2, 2, A) formulation.

**Figure 8:**
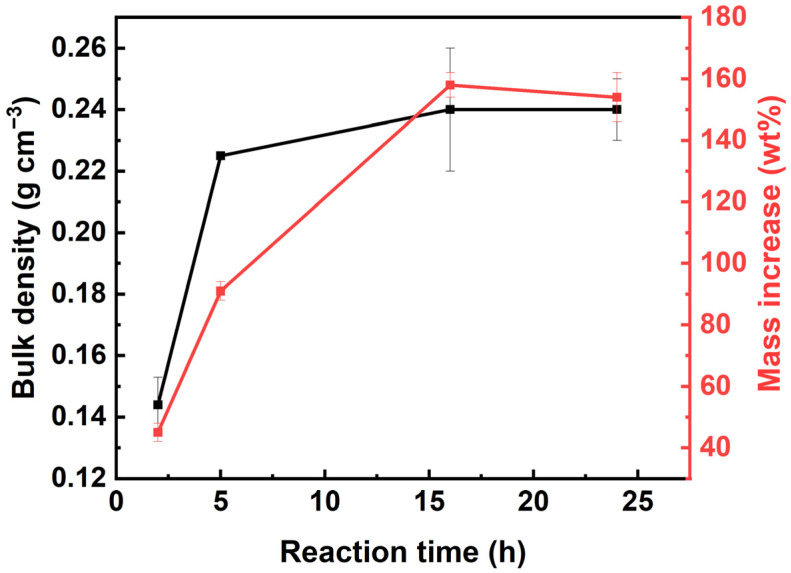
Evolution of composite aerogel density (in black) and mass (in red) as a function of MTMS deposition reaction time for (2, 2, A) formulation.

The evidence of pectin hydrophobization, for all reaction times used, is confirmed by ATR-FTIR spectroscopy (Figure S2). Two hours was sufficient time for the reaction to occur. The intensity of the broad band at 3400 cm^−1^ (Figure S2), assigned to the stretching vibrations of –OH groups of the pectin chain and water molecules (see also Figure 3), decreased with the increase in CVD time due to the interactions of pectin with the silica-based polymer and the reduction in water content inside the composite materials’ matrix after hydrophobization. The evolution of the composite aerogel properties as a function of reaction time is presented in Figure 8 for the formulation (2, 2, A) and summarized in Table S2 for other formulations. MTMS deposition resulted in a progressive increase in density, reaching a plateau value after 16 h of reaction (Figure 8). The reason for the density increase is a significant increase in sample mass, up to 150–160% (Figure 8 and Table S2); the volumetric shrinkage during reaction was not very high: within 30–40% (Table S2). The reaction time should be as short as possibles to avoid a too high increase in the final aerogels’ bulk density. The BET surface area of the composite aerogels was lower than that of the neat pectin aerogels prepared in the same conditions and was in the range of 120–172 m^2^ g^−1^ (Table S1).

**Figure 9:**
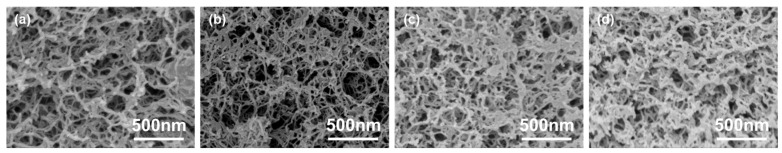
SEM images of (**a**) neat pectin aerogel (formulation 2, 2, A) and of the corresponding composite aerogels after MTMS deposition during (**b**) 2 h, (**c**) 5 h, and (**d**) 16 h.

The morphology of composite aerogels after different reaction times is presented in Figure 9. Comparing the nanostructure of neat pectin aerogels with the corresponding composite aerogels, it seemed that as the reaction time increased, the final material possessed a denser network, as expected from Figure 8. No silica-based particles were observed for reaction times up to 16 h.

**Figure 10:**
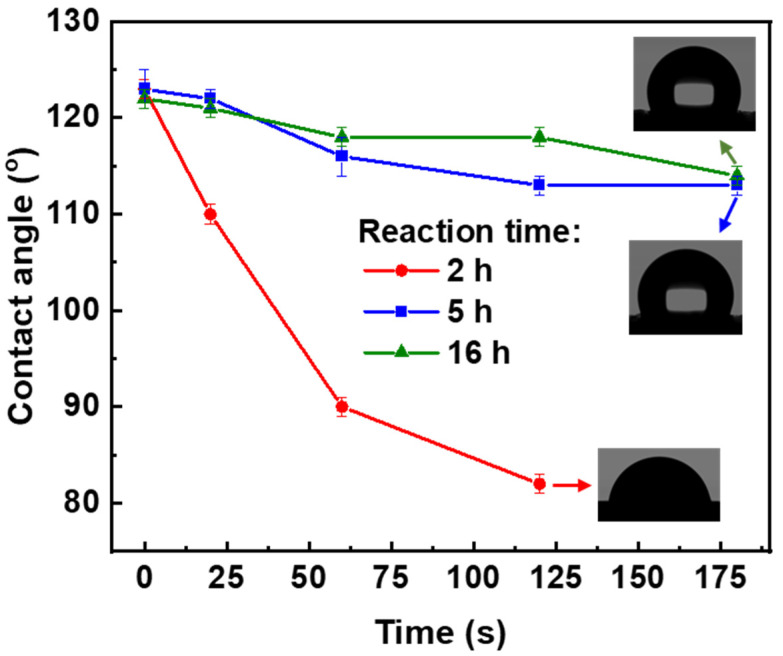
Water contact angles as a function of time for composite pectin aerogels (formulation 2, 2, A) for 2 h, 5 h, and 16 h reaction time.

The effect of the reaction time on the hydrophobicity of the composite aerogels was studied by water contact angle measurements (Table S2 and Figure 10 for the formulation (2, 2, A)). For all reaction times, the modified aerogels had high “immediate” contact angles (around 120°), which was similar to the composite aerogels obtained after 24 h of MTMS deposition (Figure 7). Exploration of the contact angle fluctuation in time (Figure 10) revealed that for short reaction times (i.e., 2 h), the contact angle decreased with water droplets being completely absorbed after three minutes. For longer reaction times (5 h or 16 h), the contact angle remained nearly stable, with minor variations, and after three minutes, no adsorption was observed. This investigation concluded that in order to achieve the stable hydrophobization of pectin aerogels, the reaction time needs to be greater than 5 h. 

**Figure 11:**
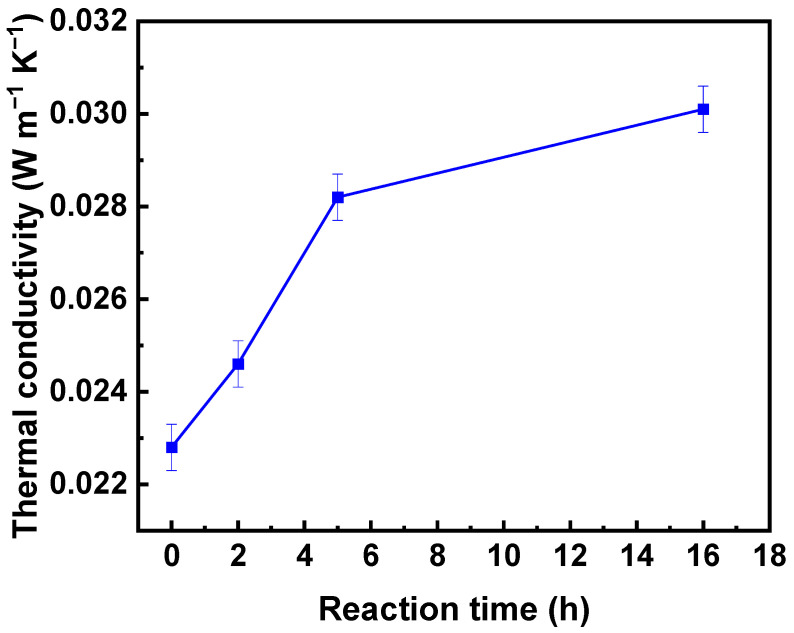
Thermal conductivity as a function of MTMS deposition reaction time for (2, 2, A) formulation.

As far as other formulations, (2, 3, A), (3, 2, A) and (3, 3, A) possess rather high (for aerogels) density values (0.31–0.57 g cm^−3^, Table S1); the formulation (2, 2, A) was selected for a further analysis of the material’s thermal conductivity (Table S1). As anticipated, the thermal conductivity of the composite aerogels increased in comparison with the corresponding neat reference counterpart (conductivity 0.0216 W m^−1^ K^−1^) as a consequence of the density increase, from 0.0246 W m^−1^ K^−1^ for 2 h reaction time to 0.0282 W m^−1^ K^−1^ for 5 h and to 0.0301 W m^−1^ K^−1^ for 16 h (Figure 11 and Table S1).

### 3.4. Aging Studies of Neat Pectin and Composite Aerogels

The aging of neat pectin aerogels and composite aerogels was monitored in a climatic chamber conditioned at constant 25 °C and relative humidity of 80%. These conditions were selected, as 25 °C is the standard reference ambient temperature, and a relative humidity of 80% is considered an “extreme” case.

**Figure 12:**
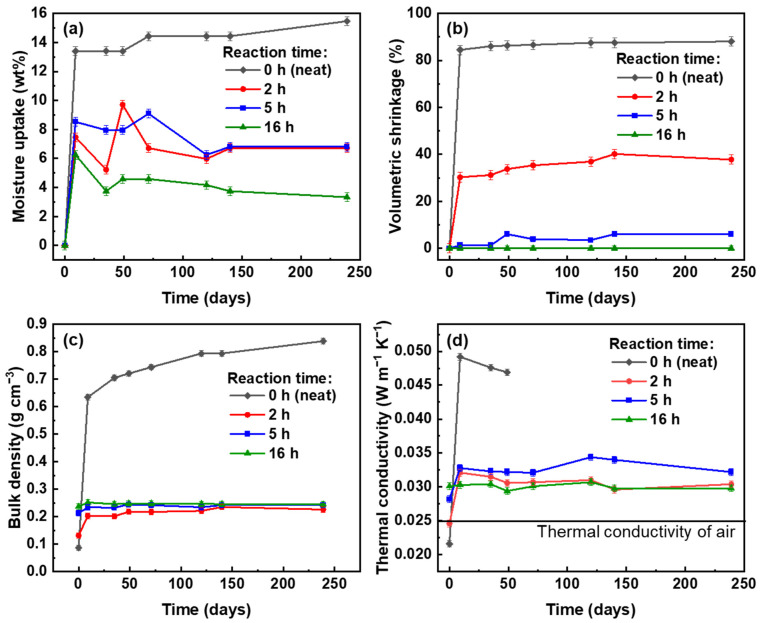
(**a**) Moisture uptake, (**b**) volumetric shrinkage, (**c**) bulk density, and (**d**) thermal conductivity as a function of residence time in the climatic chamber for neat pectin aerogels (formulation (2, 2, A) and the corresponding composite aerogels made after 2 h, 5 h, or 16 h MTMS deposition.

The volumetric shrinkage, mass increase, density and thermal conductivity evolution as a function of aging time are presented for different durations of MTMS deposition, focusing on (2, 2, A) formulation (Figure 12); some results for other formulations are also shown in Tables S2–S5. Under these high relative humidity conditions, neat pectin aerogels underwent significant changes during the first half-day (Tables S3–S6): the volumetric shrinkage was around 70–80% and the moisture uptake was around 12–18 wt%. Even though the content of water vapors adsorbed by the material was rather low, the huge shrinkage led to a high increase in bulk density. For example, for the formulation (2, 2, A), the initial density (0.087 g cm^−3^) increased to around 0.6 g cm^−3^ after 7 days (Figure 12c). This change in density resulted in high thermal conductivity values that were more than twice as large as the initial ones, from 0.022 to 0.050 W m^−1^ K^−1^ (Figure 12d). After one week and up to 240 days in the climatic chamber, neat pectin aerogels exhibited minor variations of shrinkage and moisture content, and so did the bulk density and thermal conductivity (Figure 12).

The properties of composite aerogels strongly depend on the duration of MTMS deposition (Figure 12). Moisture uptake was rather similar and below 9 wt% for the composite aerogels with different hydrophobization times (Figure 12b). Nevertheless, the volumetric shrinkage was higher for lower reaction times and was zero for the reaction time of 16 h (Figure 12a). A similar result was recorded for other formulations (Tables S3–S6). Bulk density doubled for the composite aerogel (formulation (2, 2, A)) made with 2 h of MTMS deposition, but it remained constant for the samples of 5 and 16 h (Figure 12c). It can be concluded that the network collapse and not the mass increase due to the adsorption of water vapors induced the variation in the aerogels’ density. 

A longer duration of MTMS deposition resulted in the elevated amount of hydrophobic silica-based polymer deposited on the pectin fibers, resulting in lower aerogel shrinkage in moist environments, in particular, for 5 and 16 h of reaction. At least two reasons can explain this result: (i) MTMS is hydrophobic and “protects” pectin from water adsorption, and (ii) thicker pore walls reinforce the solid backbone and increase its capacity to withstand the capillary stresses that were present during the condensation of water vapors. As a result, the nanostructure of the composite aerogels remained intact even though there was a certain proportion of moisture inside the material. Thermal conductivity measurements with time revealed that there was an increase by 0.005–0.007 W m−1 K−1 from the initial value for a shorter duration of MTMS deposition (2 h or 5 h). For a longer duration of MTMS deposition (16 h), the thermal conductivity of the composite aerogels underwent no change for 240 days.

## 4. Conclusions

Pectin aerogels were prepared, and the evolution of their properties as a function of time at 25 °C and 80 RH% was investigated with the focus on thermal conductivity. Various formulations were tested: pectin concentration 2 and 3 wt%, solution pH 2 and 3, and non-solvent ethanol and acetone. Neat pectin aerogels are thermal superinsulating materials with the lowest conductivity around 0.015 W m^−1^ K^−1^. As the adsorption and condensation of water vapors neat pectin aerogels shrink, the density and thermal conductivity significantly increase.

In the view of “protecting” pectin aerogels from aging, the hydrophobization of their solid skeleton with MTMS was performed via CVD. The influence of reaction duration, from 2 to 24 h, on the composite pectin aerogel’s properties was investigated. The longest reaction duration resulted in a high increase in aerogel density and formation of non-porous silica beads inside pectin aerogels. The 2 h reaction was not sufficient for pectin aerogel hydrophobization, as the water contact angle decreased in time and the thermal conductivity increased by 0.013 W m^−1^ K^−1^. The 16 h reaction was shown to be optimal, as no aerogel volumetric shrinkage occurred at 80 RH%, the contact angle was around 115°, and the density and thermal conductivity (around 0.030 W m^−1^ K^−1^) were stable for the whole duration of the testing period (i.e., 8 months). MTMS CVD on pectin aerogel was demonstrated to be an efficient way of pectin aerogels’ properties stabilization at high relative humidity, 80%. Even if the thermal conductivity is increased compared to the reference neat pectin aerogels, hydrophobized pectin aerogels still possess low thermal conductivity and stability in high humidity environments, which make them excellent candidates as sustainable materials for thermal insulation.

## Figures and Tables

**Table 1:** Nomenclature and properties of neat pectin aerogels.

*C_pectin_* (wt%)	pH	Non-Solvent	Nomenclature	Bulk Density, *ρ_b_* (g cm^−3^)	Porosity, ∏ (%)	BET Specific Surface Area (m^2^ g^−1^)	Thermal Conductivity, *λ* (W m^−1^ K^−1^)
2	2	ethanol	(2, 2, E)	0.050 ± 0.001	97 ± 1	322 ± 10	0.0216
2	2	acetone	(2, 2, A)	0.038 ± 0.002	98 ± 1	308 ± 16	0.0228
2	3	ethanol	(2, 3, E)	0.073 ± 0.001	91 ± 1	462 ± 28	0.0169
2	3	acetone	(2, 3, A)	0.080 ± 0.001	94 ± 1	433 ± 25	0.0148
3	2	ethanol	(3, 2, E)	0.065 ± 0.003	96 ± 1	567 ± 11	0.0212
3	2	acetone	(3, 2, A)	0.059 ± 0.001	93 ± 1	556 + 20	0.0202
3	3	ethanol	(3, 3, E)	0.11 ± 0.01	93 ± 1	475 ± 32	0.0234
3	3	acetone	(3, 3, A)	0.083 ± 0.001	96 ± 1	439 ± 8	0.0152

**Table 2:** Composition of neat pectin aerogel, formulation (2, 2, A), and of the internal area of the corresponding composite aerogel (reaction duration 24 h) as defined from EDX analysis.

Element	Neat Pectin Aerogel			Composite Pectin Aerogel		
	Mass (%)	Normalized Mass (%)	Atom (%)	Mass (%)	Normalized Mass (%)	Atom (%)
**C**	47.67	47.67	55.34	11.74	30.44	42.52
**O**	50.08	50.08	43.65	11.64	32.41	32.41
**Si**	0.56	0.56	0.28	15.18	24.07	24.07
**Na**	0.54	0.54	0.33	0	0	0
**K**	0.62	0.62	0.22	0	0	0
**Ca**	0.53	0.53	0.18	0	0	0
**Total**	100	100	100	37.57	100	100

## Data Availability

The original contributions presented in the study are included in the article/Supplementary Materials, further inquiries can be directed to the corresponding authors.

## Supplementary Materials

The following supporting information can be downloaded at https://www.mdpi.com/article/10.3390/polym16121628/s1, Figure S1: Properties of neat pectin aerogels, Figure S2: ATR-FTIR spectra for neat pectin aerogel and MTMS–pectin composite aerogels for different chemical treatment duration, Table S1: Volumetric shrinkage of neat pectin aerogels during various fabrication steps, Table S2: Selected material properties of hydrophobically modified pectin aerogels, Table S3: Properties of composite aerogels made from (2, 2, A) pectin aerogels after different reaction times of MTMS deposition (0, 2, 5 and 16 h) after aging in a conditioned climatic chamber at 25 °C and 80 RH%, Table S4: Properties of composite aerogels made from (2, 3, A) pectin aerogels after different reaction times of MTMS deposition (0, 2, 5 and 16 h) after aging in a climatic chamber at 25 °C and 80 RH%, Table S5: Properties of composite aerogels made from (3, 2, A) pectin aerogels after different reaction times of MTMS deposition (0, 2, 5 and 16 h) after aging in a climatic chamber at 25 °C and 80 RH%, Table S6: Properties of composite aerogels made from (3, 3, A) pectin aerogels after different reaction times of MTMS deposition (0, 2, 5 and 16 h) after aging in climatic chamber at 25 °C and 80 RH%.
