# Supplementary material for: Hydrophobic Modification of Pectin Aerogels via Chemical Vapor Deposition
Source: Polymers (Basel). 2024 Jun 8;16(12):1628. doi: 10.3390/polym16121628 (PMC11207865; doi:10.3390/polym16121628)
Supplement: Supplementary file 1 [file polymers-16-01628-s001.zip › polymers-3016671-supplementary.pdf]

SUPPORTING INFORMATION

Hydrophobic modification of pectin aerogels via chemical vapor deposition

Eleni Effraimopoulou [1,2], Julien Jaxel [1], Tatiana Budtova [2] *, and Arnaud Rigacci [1] *

[1]MINES Paris, PSL University, Centre for Processes, Renewable Energy and Energy Systems (PERSEE), 06904 Sophia Antipolis, France

[2]MINES Paris, PSL University, Centre for Materials Forming (CEMEF), UMR CNRS 7635, 06904 Sophia Antipolis, France

Table S1. Volumetric shrinkage of neat pectin aerogels during various fabrication steps.

Nomenclature	Volumetric shrinkage during solvent exchange (%)	Volumetric shrinkage during drying (%)	Total volumetric shrinkage (%)
(2, 2, E)	14 ± 1	61 ± 1	65 ± 3
(2, 2, A)	25 ± 2	58 ± 2	73 ± 5
(2, 3, E)	-4 ± 6	82 ± 1	79 ± 4
(2, 3, A)	21 ± 5	76 ± 4	82 ± 3
(3, 2, E)	13 ± 3	59 ± 3	64 ± 3
(3, 2, A)	23 ± 2	57 ± 4	67 ± 2
(3, 3, E)	-11 ± 3	78 ± 2	75 ± 3
(3, 3, A)	8 ± 4	66 ± 1	72 ± 4

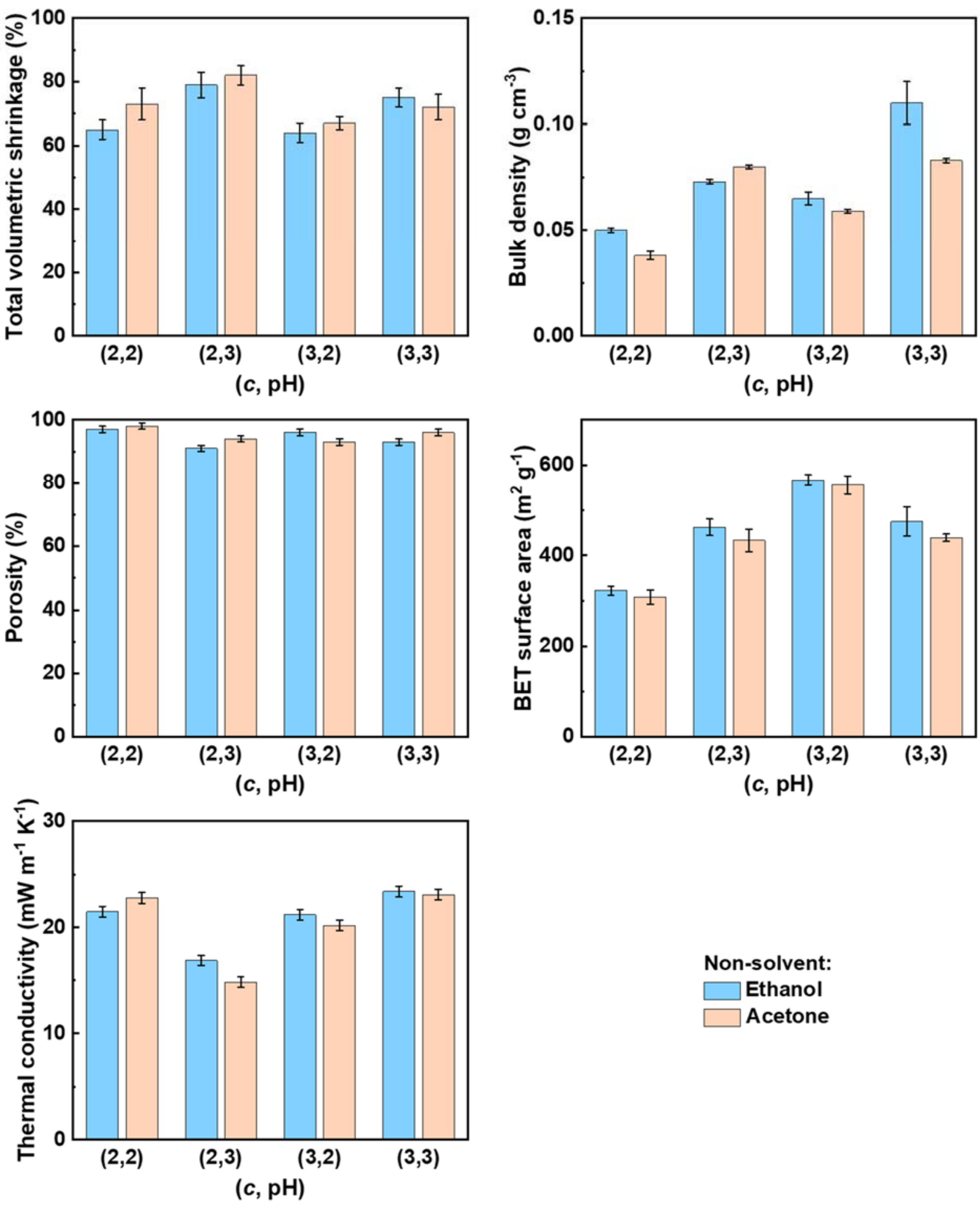

Figure S1. Properties of reference neat pectin aerogels.

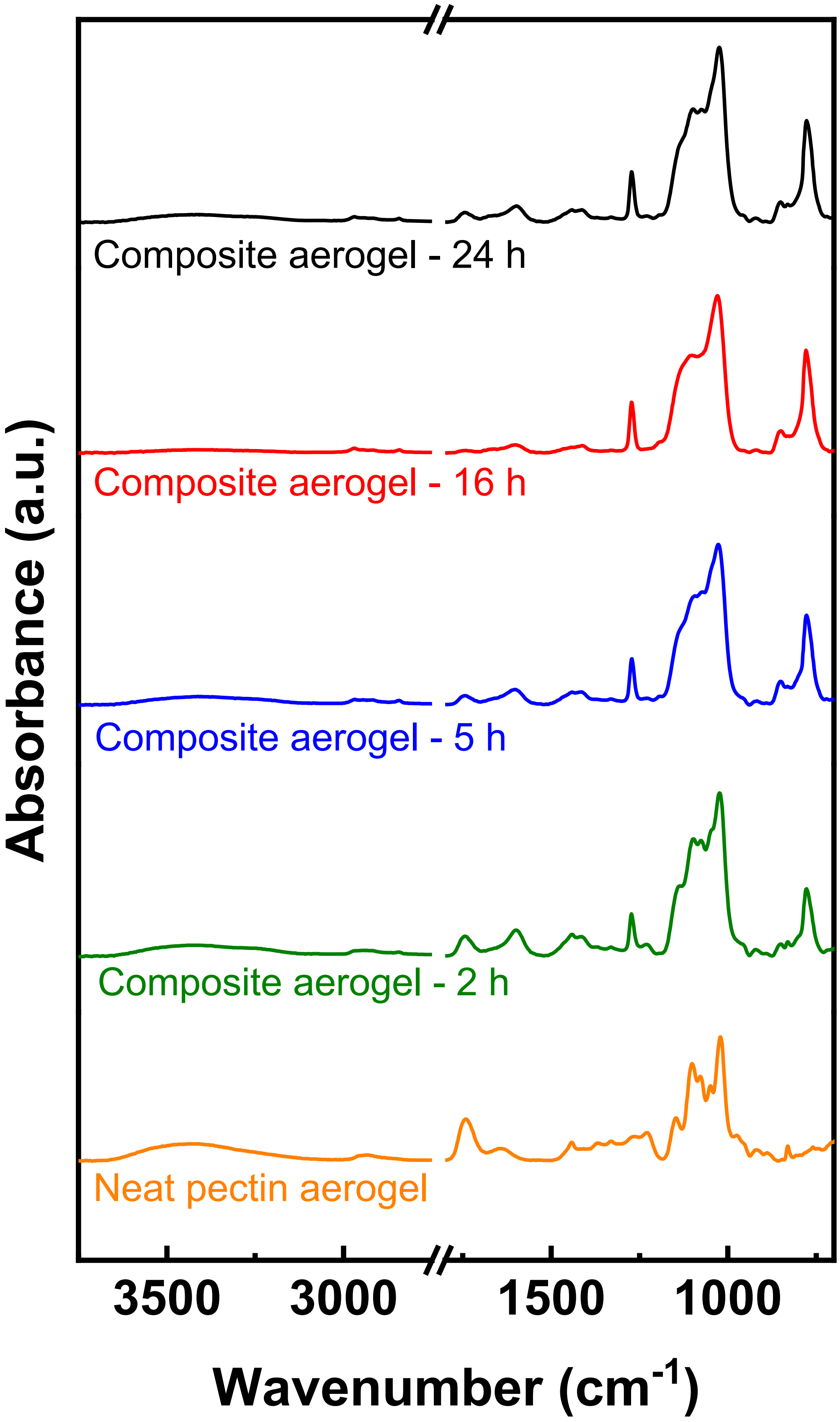

Figure S2. ATR-FTIR spectra of neat pectin aerogel and MTMS-pectin composite aerogels for different chemical vapour deposition duration.

Table S2. Selected material properties of hydrophobically modified pectin aerogels.

c_{pectin} (wt%)	pH	Non-solvent	reaction duration (h)	Volumetric shrinkage during hydrophobisation (%)	Mass increase during hydrophobisation (wt%)	Bulk density, ρ_b (g cm^{-3})	BET specific surface area (m^2 g^{-1})	Contact angle (°)	Thermal conductivity, λ (W m^{-1} K^{-1})
2	2	acetone	0	n.a.	n.a.	0.038	308	n.a.	0.0228
			2	6	45 ± 3	0.144	172	123	0.0246
			5	0	91 ± 3	0.225	130	123	0.0282
			16	29	158 ± 4	0.240	120	122	0.0301
			24	18	154 ± 8	0.240	155	118	n.m.
2	3	acetone	0	n.a.	n.a.	0.080	433	n.a.	0.0148
			5	48	90 ± 2	0.434	n.m.	n.m.	n.m.
			16	30	145 ± 2	0.462	n.m.	n.m.	n.m.
			24	28	157 ± 1	0.403	156	128	n.m.
3	2	acetone	0	n.a.	n.a.	0.059	556	n.a.	0.0202
			5	33	93 ± 2	0.315	n.m.	n.m.	0.0336
			16	24	184 ±2	0.410	n.m.	n.m.	0.0395
			24	19	155± 2	0.295	152	118	n.m.
3	3	acetone	0	n.a.	n.a.	0.083	439	n.a.	0.0152
			5	41	83 ± 2	0.373	n.m.	n.m.	n.m.
			16	40	178 ±2	0.546	n.m.	n.m.	n.m.
			24	23	147 ± 1	0.373	169	121	n.m.

Table S3. Properties of composite aerogels made from (2, 2, A) pectin aerogels after different reaction time of MTMS deposition (0, 2, 5 and 16 h) after aging in conditioned climatic chamber at temperature 25 °C and 80 RH%.

Reaction time (h)	$t_{climatic\ chamber}$ (days)	Moisture uptake (wt%)	Volumetric shrinkage (%)	Bulk density (g cm^{-3})	λ (W m^{-1} K^{-1})
0	0	n.a.	n.a.	0.087	0.0216
	7	13	84	0.634	0.0492
	35	13	86	0.705	0.0476
	50	13	86	0.721	0.0469
	70	14	87	0.744	n.m.
	120	14	87	0.794	n.m.
	140	14	87	0.794	n.m.
	240	15	88	0.839	n.m.
2	0	n.a.	n.a.	0.132	0.0246
	7	7	30	0.203	0.0321
	35	5	31	0.201	0.0315
	50	10	34	0.218	0.0306
	70	7	35	0.217	0.0307
	120	6	37	0.221	0.0310
	140	7	40	0.235	0.0296
	240	7	38	0.226	0.0304
5	0	n.a.	n.a.	0.213	0.0282
	7	8	1	0.234	0.0328
	35	8	1	0.233	0.0323
	50	8	6	0.244	0.032.2
	70	9	4	0.242	0.032.1
	120	6	4	0.234	0.034.4
	140	7	6	0.242	0.034.0
	240	7	6	0.242	0.032.2
16	0	n.a.	n.a.	0.237	0.0301
	7	6	0	0.252	0.0303
	35	4	0	0.246	0.0304
	50	5	0	0.248	0.0294
	70	5	0	0.248	0.0301
	120	4	0	0.247	0.0307
	140	4	0	0.246	0.0298
	240	3	0	0.245	0.0298

Table S4. Properties of composite aerogels made from (2, 3, A) pectin aerogels after different reaction time of MTMS deposition (0, 2, 5 and 16 h) after aging in climatic chamber at temperature 25 °C and 80 RH%.

t_{hydr} (h)	$t_{climatic\ chamber}$ (days)	Moisture uptake (wt%)	Volumetric shrinkage (%)	Bulk density (g cm^{-3})	λ (W m^{-1} K^{-1})
0	0	n.a.	n.a.	0.118	0.0233
	0.4	13	76	0.572	n.m.
	7	20	85	0.921	n.m.
	17	21	85	0.983	n.m.
	63	55	82	0.995	n.m.
	162	56	81	0.967	n.m.
5	0	n.a.	n.a.	0.432	n.m.
	0.4	7	0	0.430	n.m.
	7	9	8	0.513	n.m.
	17	8	0	0.456	n.m.
	63	8	3	0.480	n.m.
	162	6	0	0.457	n.m.
16	0	n.a.	n.a.	0.554	0.0602
	0.4	4	0	0.553	0.0650
	7	5	0	0.570	0.0614
	17	4	0	0.575	0.0637
	63	4	0	0.575	n.m.
	162	4	0	0.563	n.m.

Table S5. Properties of composite aerogels made from (3, 2, A) pectin aerogels after different reaction time of MTMS deposition (0, 2, 5 and 16 h) after aging in climatic chamber at temperature 25 °C and 80 RH%.

t_{hydr} (h)	$t_{climatic\ chamber}$ (days)	Moisture uptake (wt%)	Volumetric shrinkage (%)	Bulk density (g cm^{-3})	λ (W m^{-1} K^{-1})
0	0	-	-	0.107	0.0202
	0.4	17	78	0.571	0.0740
	7	18	86	0.911	n.m.
	17	19	86	0.936	n.m.
	63	19	87	0.957	n.m.
	162	20	87	0.984	n.m.
5	0	-	-	0.327	0.0336
	0.4	8	0	0.354	0.0370
	7	8	2	0.361	0.0374
	17	8	1	0.359	0.0374
	63	7	4	0.363	0.0373
	162	7	1	0.355	0.0374
16	0	-	-	0.401	0.0395
	0.4	4	0	0.413	0.0424
	7	4	0	0.412	0.0357
	17	4	0	0.417	0.0424
	63	4	0	0.407	0.0428
	162	4	0	0.410	0.0425

Table S6. Properties of composite aerogels made from (3, 3, A) pectin aerogels after different reaction time of MTMS deposition (0, 2, 5 and 16 h) after aging in climatic chamber at temperature 25 °C and 80 RH%.

t_{hydr} (h)	$t_{climatic\ chamber}$ (days)	Moisture uptake (wt%)	Volumetric shrinkage (%)	Bulk density (g cm^{-3})	λ (W m^{-1} K^{-1})
0	0	-	-	0.159	0.0231
	0.4	12	65	0.516	n.m.
	7	18	80	0.960	n.m.
	17	19	80	0.967	n.m.
	63	n.m.	85	0.947	n.m.
	162	n.m.	85	0.983	n.m.
5	0	-	-	0.471	n.m.
	0.4	7	0	0.461	n.m.
	7	9	0	0.476	n.m.
	17	9	0	0.476	n.m.
	63	8	0	0.482	n.m.
	162	8	0	0.482	n.m.
16	0	5-	-	0.591	0.0660
	0.4	4	0	0.606	0.0710
	7	6	0	0.600	0.0723
	17	9	0	0.634	0.0701
	63	5	0	0.610	n.m.
	162	5	0	0.612	n.m.
